# Postoperative Changes in Hematological, Biochemical, and Redox Status Parameters in Spinal Osteoarthritis Patients Undergoing Spinal Decompression and Stabilization Surgery

**DOI:** 10.3390/jcm14176306

**Published:** 2025-09-06

**Authors:** Milan Mirković, Jelena Kotur-Stevuljević, Jelena Vekić, Nataša Bogavac-Stanojević, Anđelka Milić, Sanja Mirković, Ankica Vujović, Marija Rakić, Tanja Lunić, Zoran Baščarević, Biljana Božić Nedeljković

**Affiliations:** 1 Faculty of Medicine, University of Belgrade, 11000 Belgrade, Serbia; drckmilan@yahoo.com (M.M.); zoran.bascarevic@iohbb.edu.rs (Z.B.); 2 Institute for Orthopedic Surgery “Banjica”, 11000 Belgrade, Serbia; 3 Department for Medical Biochemistry, Faculty of Pharmacy, University of Belgrade, 11000 Belgrade, Serbia; jkotur@pharmacy.bg.ac.rs (J.K.-S.); jelena.vekic@pharmacy.bg.ac.rs (J.V.); natasa.bogavac@pharmacy.bg.ac.rs (N.B.-S.); andelka.milic@iohbb.edu.rs (A.M.); 4 Faculty of Sport and Physical Education, University of Belgrade, 11000 Belgrade, Serbia; sanjakrcunovic@yahoo.com; 5 Clinic for Infectious and Tropical Diseases, University Clinical Center of Serbia, 11000 Belgrade, Serbia; ankica.vujovic88@gmail.com; 6 Institute of Physiology and Biochemistry “Ivan Djaja”, Faculty of Biology, University of Belgrade, 11000 Belgrade, Serbia; marija.mandic@bio.bg.ac.rs (M.R.); tanja.lunic@bio.bg.ac.rs (T.L.)

**Keywords:** spinal osteoarthritis, spinal decompression surgery, oxidative stress, inflammation, antioxidant defense, Kellgren–Lawrence index, glutathione, superoxide dismutase

## Abstract

**Background/Objectives:** Spinal osteoarthritis (sOA) is a degenerative condition marked by pain, inflammation, and restricted mobility. While surgical interventions such as spinal decompression and stabilization are common, their impact on redox status and inflammatory markers remains underexplored. This study aimed to assess the effects of surgery on clinical, hematological, biochemical, and redox parameters in patients with sOA. **Methods:** A total of 25 patients diagnosed with sOA underwent spinal decompression and stabilization surgery. Preoperative and postoperative assessments included hematological and biochemical analyses, redox status evaluation (TAS, TOS, GSH, AOPP, SOD), and inflammatory markers such as IL-6. Disease severity was graded using the Kellgren–Lawrence (K-L) system. **Results:** Postoperatively, there was a significant decrease in neutrophil count (*p* = 0.014) and AOPP levels (*p* < 0.001), with a corresponding increase in lymphocyte count (*p* = 0.016), erythrocyte count (*p* = 0.036), and IL-6 levels (*p* = 0.008). TAS levels decreased (*p* = 0.006), while enzymatic antioxidants, such as SOD increased (*p* = 0.031). Erythrocyte GSH remained low, with a non-significant postoperative decrease. Patients with higher K-L grades exhibited greater redox imbalance, with reduced preoperative GSH and elevated postoperative superoxide anion, TOS, and SOD levels. More severe cases also showed decreased postoperative erythrocyte, hemoglobin, and PTH levels, and increased TAS and AOPP levels. Factorial analysis highlighted clusters associated with oxidative stress, inflammation, and clinical performance. **Conclusions:** The results underscore the complex relationship between inflammation, oxidative stress, and recovery in sOA. These findings suggest the importance of targeted postoperative strategies to support redox homeostasis and modulate inflammation in sOA patients.

## 1. Introduction

Spinal osteoarthritis (sOA) is a degenerative joint disease affecting the spine, particularly involving the facet joints located at the posterior aspect of the vertebral column. It primarily develops due to the aging process, encompassing both mechanical and oxidative stress. The condition is marked by pain and restricted mobility, which considerably impair daily functioning [1,2].

Aging and traumatic injuries, characterized by mechanical stress, are closely associated with oxidative stress and synovial inflammation, which are pivotal in the development of osteoarthritis (OA) [3,4]. The cartilage structure is mainly composed of chondrocytes cells, embedded within an extracellular matrix (ECM) of collagen and proteoglycans. Oxidative stress arises when there is an imbalance between reactive oxygen species (ROS) production and the cellular antioxidant defense system, which includes enzymatic components such as catalases, peroxiredoxins, glutathione peroxidase, NADPH ubiquinone oxidoreductase, and superoxide dismutases (SODs), as well as non-enzymatic antioxidants like glutathione (GSH). Oxidative stress involves various pro-inflammatory mediators, including ROS such as superoxide (•O_2_^−^), hydroxyl radicals (•OH), and nitric oxide (NO), as well as hydrogen peroxide (H_2_O_2_) and peroxynitrite (ONOO^−^) [5]. Additionally, pro-inflammatory cytokines such as TNFα, IL-1β, and IL-6, which are produced by chondrocytes, synoviocytes, osteoblasts, and infiltrating immune cells, contribute to the inflammatory milieu in the synovial joint [5]. Elevated levels of pro-inflammatory mediators induce matrix-degrading proteases, which decrease ECM synthesis, and activate chondrocyte apoptosis signaling pathways. At the cellular level, oxidative stress causes damage to mitochondrial and nuclear DNA, promotes lipid peroxidation, disrupts cell signaling, and leads to epigenetic alterations in gene expression [6].

These pathological processes not only promote OA progression, but also hinder cartilage regeneration. Excessive ROS in the microenvironment of the intervertebral disk results in chronic inflammation characterized by pain and impaired movement [3,7]. Therefore, maintaining redox homeostasis is crucial for reducing the severity of OA and mitigating disease progression.

In our previous study [8], we investigated the effects of lower back surgery, e.g., spinal decompression with vertebra stabilization, on upper limb maximum strength and found a significant improvement in lower limb functionality but no change in handgrip strength. A trend towards improved quality of life was observed, particularly in female. These results indicate the effectiveness of surgery for sOA and suggest the need for personalized treatment approaches. The aim of our current study is focused on assessing hematological, biochemical and redox status parameters before and after surgery to further elucidate the impact of surgical intervention on the patients’ overall health and physiological status.

## 2. Materials and Methods

### 2.1. Biochemical Analysis

All patients followed a standardized rehabilitation program and received standard perioperative pharmacological care, including non-steroidal anti-inflammatory drugs and vitamin B complex supplementation. Postoperative samples were collected at patient follow-up visits, which varied individually (on average 5 months post-surgery). Routine hematological and biochemical analysis was performed in the Clinical Chemistry Laboratory of the “Banjica” hospital using routine commercial methods with the following analyzers: immunochemistry analyzer Cobas e411 (Roche Diagnostic GmbH, Mannheim, Germany), automated coagulometer BCS XP (Siemens, Marburg, Germany), biochemical analyzer Olympus AU 480 (Beckman Coulter Ireland Inc., Naas, Ireland), hematology counter ADVIA 2120i (Siemens, Tarrytown, NY, USA), and hematology counter Sysmex XN450 (Kobe, Japan).

Redox status parameters were performed in the Laboratory of the Department of Medical Biochemistry, Faculty of Pharmacy, University of Belgrade, using validated methods. Advanced oxidation protein products (AOPP) were determined according to the Witko–Sarsat method, using a reaction with glacial acetic acid and potassium iodide [9]. For the method calibration, chloramine T was used in the 10–100 μmol/L concentration range.

Prooxidative–antioxidative balance (PAB) was determined by a modified PAB test using 3,3′,5,5′-tetramethylbenzidine as a chromogen and a mixture of different uric acid and H_2_O_2_ concentration ranges [10]. Spectrophotometric method, based on the reaction between albumin and cobalt chloride, was used for IMA determination [11]. Total oxidative status (TOS) was measured by a spectrophotometric method using o-dianisidine, and total antioxidative status (TAS) was measured by a spectrophotometric method using ABTS as a chromogen [12].

Plasma SOD activity was measured according to the method of Misra and Fridovich [13], using the inhibition of epinephrine autooxidation caused by enzyme. Serum PON1 activity was measured kinetically using paraoxon as substrate (Chem Service, West Chester, PA, USA), as published by Richter and Furlong [14]. Total sulfhydryl groups in plasma were measured by the Ellman method [15], using DTNB (dinitrodithiobenzoic acid) as a reagent and reduced glutathione as a standard. Reduced glutathione was assayed according to the method of Jollow et al. [16]. The method is based on the reaction between GSH in a sample and 0.4% 5,5′-dithiobis-2-nitrobenzoic acid, after deproteinization with 1.0 mL of 4% sulfosalicylic acid.

### 2.2. Statistical Analysis

Depending on the type of variable and the normality of distribution, data were presented as *n* (%), or median (25th–75th percentile). The statistical tests used for hypothesis testing included a Wilcoxon paired test, a Kruskal–Wallis non-parametric ANOVA, and a Mann–Whitney U test as a post hoc test, chi-square test, and Fisher’s exact test. Factorial analysis was performed as principal component analysis with varimax rotation. Statistical hypotheses were tested at a significance level (alpha) of 0.05. All statistical procedures were performed using IBM SPSS Statistics 24 (IBM Corporation, Armonk, NY, USA).

### 2.3. Sample Size Calculation

The minimum required sample size to detect an effect size of 0.33 in a repeated measures ANOVA (2 time points) for parameters related to oxidative stress, inflammation, and active bone remodeling, with a significance level of 0.05, power of 0.95, and an assumed correlation of 0.5 between repeated measures, was calculated to be 21 patients. The estimated effect size was based on a medium effect size according to Cohen’s conventions (f = 0.33; η^2^ = 0.098), corresponding to an explained-to-residual variance ratio of ~0.11. To account for potential data loss during the study (missing or unusable data), this number was increased by 20%, resulting in a final planned minimum sample size of 25 participants.

## 3. Results

### 3.1. Clinical and Demographic Characteristics of Patients

This study comprised 25 patients (Table 1), with a predominance of women (64%, *n* = 16). The mean age was 59.1 ± 9.9 years, and the majority were obese (60%). Disease severity, as assessed by the Kellgren–Lawrence (K-L) index, which grades osteoarthritis from stage 0 (no OA) to stage 4 (severe OA), showed that most patients were classified as stage 3 (52%), followed by stage 4 (36%). The Pfirrmann grading system showed that patients were mainly categorized as grade 4 (56%) and grade 5 (44%), reflecting the extent of disk degeneration.

### 3.2. Changes in Hematological and Biochemical Parameters Post-Surgery

#### Inflammatory and Hematological Markers

Postoperative analysis of hematologic parameters showed that most values remained within the reference ranges; however, some significant changes were observed. The neutrophil count decreased significantly (*p* = 0.014), while the lymphocyte count increased (*p* = 0.016), indicating a change in the immune response after surgery. In addition, the erythrocyte count showed a significant postoperative increase (*p* = 0.036), possibly due to an improved oxygen transport capacity. A significant increase in D-dimer values (*p* < 0.001), which exceeded the upper reference limit, indicates sustained coagulation activity in the postoperative period. In contrast, CRP levels did not change significantly (*p* = 0.166) and remained stable within reference ranges, while neutrophil and lymphocyte numbers fluctuated, leading to changes in the neutrophil-to-lymphocyte ratio. Also, no significant differences were observed in erythrocyte sedimentation rate (ESR), hemoglobin concentration or platelet count, indicating an overall stable hematologic profile after surgery (Table 2).

### 3.3. Changes in Redox Status Post-Surgery

#### 3.3.1. Serum Redox Markers

As shown in Table 3, the analysis of oxidative stress markers in serum revealed significant postoperative changes. A significant decrease in advanced oxidation protein products (AOPP) (*p* < 0.001) was observed, indicating less protein oxidation after surgery. However, an increase in ischemia-modified albumin (IMA) (*p* = 0.024) and prooxidant–antioxidant balance (PAB) (*p* < 0.001) indicated persistent oxidative stress, possibly related to postoperative inflammation. Total antioxidant status (TAS) decreased significantly (*p* = 0.006), suggesting depletion of systemic antioxidant capacity in the postoperative period. This decrease was accompanied by a significant increase in enzymatic antioxidants, including superoxide dismutase (SOD) (*p* = 0.031) and paraoxonase 1 (PON1) (*p* = 0.002). The increase in these antioxidant enzymes likely represents a compensatory response to prolonged oxidative stress. Despite these changes, the TAS/TOS ratio remained unchanged and well below the reference values, suggesting that full redox homeostasis had not yet been restored after surgery (Table 3).

#### 3.3.2. Erythrocyte Redox Markers

Postoperative analysis of redox markers in the erythrocytes (Table 4) revealed that TOS showed a decreasing trend (*p* = 0.077), although this change was not statistically significant. Erythrocyte GSH was low relative to the laboratory reference interval at both time points and showed a non-significant decrease postoperatively (0.475 → 0.418 μmol/g Hb; *p* = 0.094). In addition, superoxide anion (•O_2_−), SOD activity and sulfhydryl groups (SHG) remained elevated in the erythrocytes compared to reference values.

### 3.4. Association Between Disease Severity and Biochemical Parameters

#### Kellgren-Lawrence Index (K-L)

**Figure 1:**
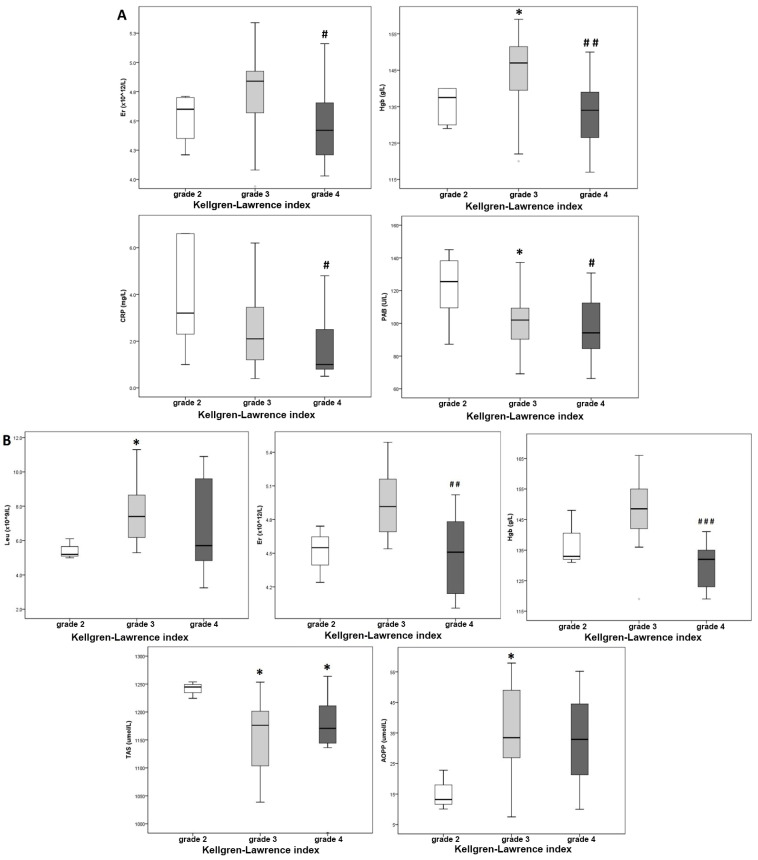
Hematological, biochemical and redox status parameters according to Kellgren–Lawrence index before (**A**) and after (**B**) surgery; * *p* < 0.05 vs. K-L index = grade 2, #, ##, ### *p* < 0.05, 0.01, 0.001 vs. K-L index = grade 3.

Before surgery, patients with higher K-L grades had lower hemoglobin and erythrocyte counts, indicating anemia-like features in more severe OA cases. Interestingly, CRP and PAB levels were unexpectedly low in these patients. Postoperatively, patients with higher K-L scores had significantly increased leukocyte counts, while erythrocyte counts and hemoglobin levels remained lower in the most severely ill patients. Additionally, in the postoperative period, a comparison between the three patient groups based on initial K-L index values revealed a significant increase in TAS and AOPP levels in patients with more severe OA (Figure 1).

**Figure 2:**
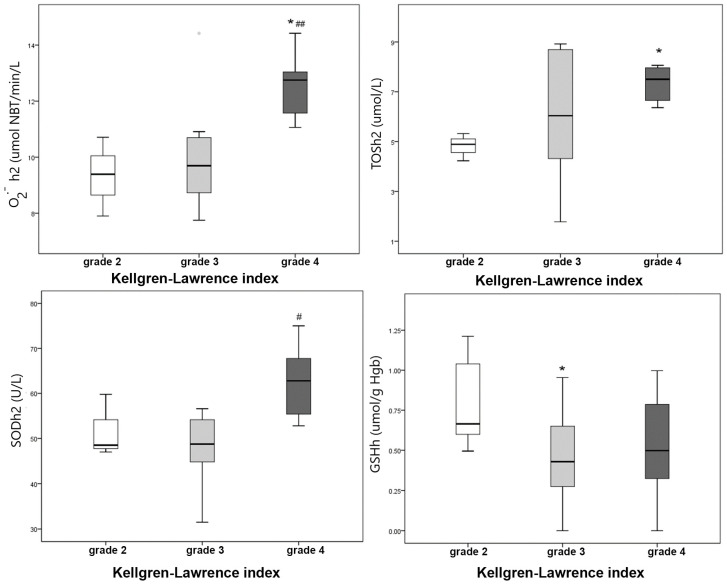
Erythrocytes’ redox status parameters according to Kellgren–Lawrence index before (GSH) and after surgery (•O_2_^−^, TOS and SOD); h—hemolysate before surgery, h2—hemolysate after surgery; * *p* < 0.05 vs. K-L index = grade 2, #, ## *p* < 0.05, 0.01vs. K-L index = grade 3.

Further analysis of oxidative stress markers in erythrocytes revealed additional trends. Patients with higher K-L levels had significantly lower GSH levels before surgery, suggesting more pronounced oxidative stress in patients with severe disease. However, after surgery, superoxide anion (•O_2_^−^), TOS and SOD activity were significantly higher in patients with the highest K-L index, suggesting that oxidative stress persisted in these individuals after surgery (Figure 2).

### 3.5. Factorial Analysis of Hematological, Biochemical, and Redox Parameters

The factorial analysis revealed four main clusters, which accounted for 57% of the total variance (Table 5). The first factor, “Redox factor”, consisted of TAS, IMA, TOS and SHG, reflecting oxidative stress and antioxidant capacity. The second factor, “Red blood cell and clinical performance factor”, includes hemoglobin, erythrocytes, 6 min walk test, and handgrip strength, reflecting the relationship between red blood cell function and physical performance. The third factor, “Inflammation/coagulation factor”, consisted of fibrinogen, neutrophils, lymphocytes and platelets, which indicates the interaction of inflammation and coagulation in OA patients.

## 4. Discussion

This study investigated the clinical, biochemical and redox status changes in patients with sOA after spinal decompression and vertebral stabilization surgery. The results revealed several important findings, including postoperative changes in hematologic and inflammatory markers, shifts in oxidative stress levels, and notable correlations between disease severity and oxidative stress parameters.

The hematologic parameters remained largely within the reference values, with notable exceptions. The decrease in neutrophil count and increase in lymphocyte count observed postoperatively could indicate a shift from an acute inflammatory response to a more regulated immune status. This observation is consistent with studies showing that a higher ratio of neutrophils to lymphocytes is associated with greater severity of osteoarthritis, emphasizing the role of systemic inflammation in disease progression [17,18,19].

The observed changes in oxidative stress markers provide valuable insights into the redox balance during postoperative recovery. A significant decrease in AOPP concentration after surgery indicates less oxidative protein damage, which is consistent with previous reports that AOPP concentration in human synovial fluid is positively associated with the severity of OA [20]. This study showed that elevated AOPP levels contribute to cartilage degeneration in OA, highlighting the role of oxidative stress in disease progression. The decrease in AOPP levels after surgery observed in the present study may indicate a reduction in oxidative damage, which could be associated with improved joint function and a decrease in inflammation.

On the other hand, contrary to expectations, IMA and PAB levels increased, indicating that oxidative stress persisted despite the surgery. In addition, surgical trauma itself can lead to a transient increase in oxidative markers. The increase in IMA observed postoperatively is consistent with its role as a marker of ischemia and oxidative stress, as elevated levels have been reported after arthroscopic knee surgery [21]. Similarly, ischemic conditions during major arterial and orthopedic surgeries have been associated with increased IMA levels, further supporting its link to postoperative oxidative stress [22,23]. While there is limited data on PAB levels in OA surgery, PAB is known to fluctuate in response to inflammation and tissue remodeling [24]. Its postoperative increase could reflect an inflammatory phase after surgery.

The decrease in TAS is another interesting result. A decrease in TAS indicates antioxidant depletion, which is often observed in chronic inflammation and oxidative stress [25,26]. The simultaneous increase in enzymatic antioxidants (SOD and PON1) appears to be a compensatory response, indicating an attempt to restore the redox balance by increasing the activity of antioxidant enzymes [6,27,28]. These observations are broadly consistent with reports in knee and hip osteoarthritis, as well as in other orthopedic surgical cohorts, where surgery acts as a metabolic and inflammatory stressor, but also initiates adaptive antioxidant responses. For example, the decrease in TAS we observed after spinal surgery mirrors findings in knee OA patients after hip arthroplasty, where surgery was shown to trigger systemic oxidative stress [29]. For instance, a 21-day rehabilitation program post-hip or -knee arthroplasty led to significant increases in total SOD, reflecting enhanced antioxidant defense and corresponded with improved 6 min walk test performance [30]. Although the GSH concentration in the erythrocytes showed a non-significant trend towards lower values postoperatively, this could be due to a still-incomplete recovery of the antioxidant defenses. This pattern is consistent with findings in patients with OA and rheumatoid arthritis, in whom decreased GSH levels in erythrocytes were found, indicating impaired antioxidant capacity compared to healthy controls [31,32]. These findings highlight the complexity of redox homeostasis in erythrocytes after surgery and suggest the need for further research into interventions that could support antioxidant recovery.

Patients with higher Kellgren–Lawrence scores had lower hemoglobin and erythrocyte levels before surgery, which could indicate a correlation between the severity of sOA and altered hematologic parameters. While anemia is well documented in chronic inflammatory diseases such as rheumatoid arthritis [33], its connection with OA is less well documented. In contrast to rheumatoid arthritis, OA is not primarily considered a systemic inflammatory disease, which may explain the lack of a clear link between chronic inflammation and anemia in OA patients.

The lower CRP and PAB levels observed in patients with severe Kellgren–Lawrence (K-L) scores may indicate that the inflammation in advanced sOA is more localized and less detectable systemically. However, studies have found conflicting trends in CRP levels in progressive OA. Some results suggest that plasma concentrations of monomeric C-reactive protein (mCRP) are significantly higher in patients with advanced OA (K-L grade 4) than in patients with K-L grade 3, suggesting that mCRP reflects disease severity rather than a decrease in systemic inflammation [34]. Other study has reported that higher high-sensitivity CRP (hs-CRP) levels are associated with the progression of knee OA [35]. The discrepancy between these findings and the current results could be due to differences in the specific CRP isoforms measured or differences in patient cohorts and disease phenotypes. These results emphasize the complex interplay between systemic inflammation and local joint pathology in OA progression. In our study, CRP remained stable at follow-up, as sampling was performed months after surgery, well beyond the typical acute postoperative peak [36]. At this point, CRP had likely normalized, whereas leukocyte subsets (including the shift in neutrophil to lymphocyte ratio) remained more variable, reflecting residual immunoremodulation not captured by CRP. This is consistent with the results in orthopedic patients following knee or hip replacement, in whom postoperative NLR, but not CRP, correlated with recovery parameters and postoperative pain [37].

The factorial analysis revealed the complex relationships between oxidative stress, inflammation, and bone metabolism in sOA patients. Key factors identified include redox, inflammation/coagulation, and red-blood-cell-related clinical performance, which together highlight the multifaceted nature of sOA pathophysiology and recovery post-surgery.

Notably, hematological parameters like Hgb and Er correlated with clinical performance, such as the 6 min walk test and handgrip strength, underscoring the role of red blood cell function in recovery [38]. Although blood loss, which may influence recovery, was not examined in the present study, the positive correlation between red blood cell indices and physical performance suggests that efficient oxygen transport is essential for post-surgical recovery. The inflammation–coagulation factor, including fibrinogen, neutrophils, and platelets, and its link to oxidative stress further emphasizes the role of systemic inflammation in recovery [39]. Although we did not directly correlate every redox marker with pain/mobility scores, the factor analysis indicates that functional outcomes align with hematologic redox changes, supporting their clinical relevance. Future studies should prospectively assess biomarker–outcome correlations. Overall, the factorial analysis highlights the intricate interplay between redox balance, inflammation, and red blood cell function, all of which are critical to improving recovery and clinical outcomes after surgery.

An important clinical implication of our findings is that postoperative sOA patients may remain in a state of redox imbalance despite improved mobility. Specific strategies to restore redox homeostasis could include supplementation with GSH precursors, such as N-acetylcysteine or glycine, to support endogenous glutathione synthesis [40], and a diet enriched with vitamin C [41] and polyphenols [42] to increase overall antioxidant capacity and structured exercise rehabilitation, which has been shown to increase endogenous antioxidant defenses. In addition, structured physical rehabilitation programs have been shown to improve the functional abilities of patients undergoing orthopedic procedures, such as knee or hip replacements [43,44]. The effectiveness of such interventions could be monitored by serial measurements of erythrocyte GSH, TAS/TOS, and markers of oxidative damage (AOPP, IMA), in combination with functional outcomes such as walking distance and grip strength. These approaches should be further investigated in larger, controlled studies to optimize the postoperative recovery of patients with sOA.

Several methodological limitations should be emphasized. First, our sample is modest (*n* = 25) and represents a tertiary surgical cohort enriched by obesity and advanced disease stage (88% K–L grade 3–4). This increases the internal validity for the surgical population, but limits the generalizability to earlier disease stages. Second, the absence of a non-surgical sOA or healthy control group limits our ability to disentangle surgical effects from natural disease progression. The present findings therefore reflect peri- and post-operative trajectories within surgical sOA patients rather than establishing causality. Third, although all patients underwent standardized surgery and rehabilitation, unmeasured heterogeneity in terms of pharmacological therapy and comorbidities may have influenced the responses.

In summary, despite surgical treatment, patients with advanced sOA have persistent oxidative stress and low GSH levels, associated with adaptive upregulation of antioxidant enzymes and functional improvements. These findings support the concept that targeted postoperative strategies to support redox homeostasis may optimize recovery and long-term outcomes. Future studies should include comparison groups, stratify by comorbidities and pharmacological exposures, and test specific interventions to restore redox balance in the postoperative period.

## 5. Conclusions

In conclusion, this study shows that spinal decompression and stabilization surgery leads to significant changes in hematologic, inflammatory, and redox status parameters in patients with sOA. While surgery reduces oxidative protein damage, as evidenced by decreased AOPP levels, it also improves several hematologic markers, including neutrophil and lymphocyte counts. In addition, the increase in antioxidant enzyme activity (PON1 and SOD) suggests a beneficial effect in attenuating oxidative damage. Further analysis of erythrocyte-specific markers revealed that although oxidative stress was reduced postoperatively, GSH remained persistently low relative to reference values, indicating persistent antioxidant impairment. This pattern suggests that although surgery provides some relief of oxidative damage, the redox imbalance persists at the cellular level. These findings highlight the importance of monitoring oxidative stress and inflammation after surgery and point to the potential benefits of antioxidant and anti-inflammatory interventions to aid recovery. The results obtained improved our understanding of the systemic impact of surgical treatment to oxidative stress and antioxidative system in sOA patients. Future research should investigate targeted strategies to restore redox balance and improve postoperative outcomes.

## Figures and Tables

**Table 1:** Anthropometric and clinical patients’ data.

Parameter	Number
*N*	25
Gender, female *n* (%)	16 (64)
Age (years)	59.1 ± 9.9
Obesity, no/yes, *n* (%)	10/15 (40/60)
BMI (kg/m^2^)	27.5 ± 4.7
Kellgren–Lawrence index, *n* (%)	
2	3 (12)
3	13 (52)
4	9 (36)
Pfirrmann grade, *n* (%)	
4	14 (56)
5	11 (44)

BMI—body mass index.

**Table 2:** Change in hematological and biochemical parameters in a group of osteoarthritis patients after surgery.

Parameters	Before Surgery	After Surgery	*p*	Reference Values
ESR (mm/h)	13 (10–20.0)	16 (9–20)	0.646	1–30 male 1–20 female
Leu (×10^9^/L)	7.8 (6.0–8.8)	6.4 (5.4–8.6)	0.143	3.4–9.7
Neu (×10^9^/L)	4.7 (3.8–5.5)	4.0 (3.0–5.1)	0.014	2.1–6.5
Lym (×10^9^/L)	1.8 (1.2–2.1)	1.9 (1.6–2.3)	0.016	1.2–3.4
Er (×10^12^/L)	4.56 (4.23–4.88)	4.73 (4.54–4.93)	0.036	4.43–5.72 male 3.86–5.08 female
Hgb (g/L)	139 (130–147)	137 (132–148)	0.726	138–175 male 119–157 female
Tr (×10^9^/L)	250 (226–305)	256 (224–299)	0.353	158–424
Fibrinogen (g/L)	3.50 (3.00–3.70)	3.50 (3.20–3.80)	0.772	2.1–4.0
CRP (mg/L)	2.05 (1.00–3.42)	2.30 (1.22–3.58)	0.166	0–5

ESR—erythrocytes sedimentation rate, Le—leukocytes count, Ne—neutrophils count, Ly—lymphocytes count, Er—erythrocytes count, Hgb—hemoglobin, Tr—thrombocytes count, CRP—C-reactive protein, PCT—procalcitonin, PTH—parathyroid hormone; *p* from Mann–Whitney U test.

**Table 3:** Redox status parameters change in a group of osteoarthritis patients after surgery.

Parameters	Before Surgery	After Surgery	*p*	Reference Values
AOPP (μmol/L)	57.1 (43.2–109.5)	28.6 (18.2–40.9)	<0.001	9–28
IMA (ABSU)	0.524 (0.448–1.313)	1.087 (0.745–1.334)	0.024	<0.400
PAB (U/L)	94 (87–112)	102 (90–113)	<0.001	0–80
TOS (μmol/L)	107 (95–127)	98 (93–117)	0.178	6–13
TAS (μmol/L)	1272 (1141–1349)	1182 (1140–1228)	0.006	900–1400
TAS/TOS	11.5 (9.3–14.5)	11.8 (9.7–13.3)	0.962	>100
SOD (U/L)	80 (75–88)	88 (76–98)	0.031	90–180
PON1 (U/L)	233 (127–418)	265 (142–451)	0.002	200–1080
SHG (mmol/L)	0.374 (0.265–0.530)	0.425 (0.275–0.485)	0.614	0.315–0.600
Prooxidant score	15.2 (12.3–17.9)	11.4 (10.1–14.2)	0.304	4.60 (3.64–6.14)
Antioxidant score	2.2 (1.4–2.8)	1.6 (0.7–2.0)	0.304	−0.031 (−0.906–0.784)
Oxy score	13.1 (10.2–14.9)	10.6 (9.0–12.4)	0.454	4.94 (3.21–6.19)

AOPP—advanced oxidation protein products, IMA—ischemia-modified albumin, PAB—prooxidant–antioxidant balance, TOS—total oxidant status, TAS—total antioxidant status, SOD—superoxide dismutase, PON1—paraoxonase 1, SHG—sulfhydryl groups.

**Table 4:** Redox status parameters in erythrocytes in a group of osteoarthritis patients before and after surgery.

Parameters	Before Surgery	After Surgery	*p*	Reference Values
TOS (μmol/L)	7.8 (6.8–9.0)	6.5 (4.9–8.4)	0.077	5.8–7.6
•O_2_^−^ (μmol NBT/min/L)	10.8 (9.9–12.2)	10.6 (8.9–11.7)	0.445	7.4–9.1
SOD (U/L)	56.7 (51.4–61.5)	53.2 (47.0–59.8)	0.685	5.9–8.4
SHG (mmol/L)	0.768 (0.624–0.953)	0.680 (0.595–0.762)	0.115	0.205–0.267
GSH (μmol/g Hgb)	0.475 (0.317–0.720)	0.418 (0.265–0.534)	0.094	4.4–9.8 male 4.8–10.5 female

TOS—total oxidant status, •O_2_^−^—superoxide anion, SOD—superoxide dismutase, SHG—sulfhydryl groups, GSH—reduced glutathione; *p* from Wilcoxon’s paired test.

**Table 5:** PCA-derived factors in osteoarthritis patients.

Factors	Variables Included in the Factor	Loadings of the Variables	Factor Variability, % (Total Variance: 57%)
Redox factor	TAS	−0.944	21
	IMA	0.842	
	TOS	0.791	
	SHG	0.786	
Red blood cells—patients’ clinical performance-related factor	Hgb (g/L)	0.824	20
	Er (×10^12^/L)	0.817	
	6 min walking test (m)	0.707	
	Hand grip strength	0.650	
	ESR (mm/h)	−0.600	
Inflammation–procoagulation-related factor	Fibrinogen (g/L)	0.820	16
	Neu (×10^9^/L)	0.788	
	Tr (×10^9^/L)	0.611	
	Ly (×10^9^/L)	0.578	

TAS—total antioxidant status, IMA—ischemia-modified albumin, TOS—total oxidant status, SHG—sulfhydryl groups, Hgb—hemoglobin, ESR—erythrocytes sedimentation rate; Neu—neutrophils count, Tr—thrombocytes count, Ly—lymphocytes count.

## Data Availability

Data are contained within the article.

## Abbreviations

The following abbreviations are used in this manuscript:

AOPP	Advanced oxidation protein products
BMI	Body mass index
CRP	C-reactive protein
ESR	Erythrocyte sedimentation rate
GSH	Reduced glutathione
Hgb	Hemoglobin
IL-1β	Interleukin-1 beta
IL-6	Interleukin-6
IMA	Ischemia-modified albumin
K-L	Kellgren–Lawrence
Leu	Leukocyte count
Lym (Ly)	Lymphocyte count
Neu (Ne)	Neutrophil count
NO	Nitric oxide
•O_2_^−^	Superoxide anion
OA	Osteoarthritis
ONOO^−^	Peroxynitrite
PAB	Prooxidant–antioxidant balance
PON1	Paraoxonase 1
PTH	Parathyroid hormone
ROS	Reactive oxygen species
SHG	Sulfhydryl groups
sOA	Spinal osteoarthritis
SOD	Superoxide dismutase
TAS	Total antioxidant status
TNFα	Tumor necrosis factor alpha
TOS	Total oxidant status
Tr	Thrombocyte (platelet) count
